# Response to Wolf et al.: Furthering Debate over the Suitability of Trap-Neuter-Return for Stray Cat Management

**DOI:** 10.3390/ani10020362

**Published:** 2020-02-23

**Authors:** Michael C. Calver, Heather M. Crawford, Patricia A. Fleming

**Affiliations:** Environmental and Conservation Sciences, Murdoch University, Perth, WA 6150, Australia; crawfh01@gmail.com (H.M.C.); t.fleming@murdoch.edu.au (P.A.F.)

**Keywords:** adopt, conservation ethics, neuter, wildlife

## Abstract

**Simple Summary:**

Proponents of different views over the desirability of trialling Trap-Neuter- Release (TNR) in Australia agree that Australia has a problem with stray cats, necessitating reduction in stray cat numbers to reduce impacts on wildlife, nuisance, disease transmission (including public health issues and exchange of diseases between stray cat and pet cat populations), poor welfare outcomes for stray cats, and an unacceptable emotional burden on staff required to euthanise healthy stray cats. They disagree (i) whether current measures have failed or have led to unacceptably high levels of euthanasia, (ii) whether all contributors to the debate understand TNR, (iii) whether TNR trials will reduce urban cat populations and associated problems, and (iv) whether TNR can be considered an ethical solution to the problem of cat overpopulation. Furthermore, (v) it is alleged that some contributors to the debate distribute misinformation. Although we take the position that a TNR trial is premature, as a hypothetical exercise, we recommend that any such trial should use an experimental approach to compare TNR explicitly to alternatives.

**Abstract:**

To continue dialogue over proposed Australian trials of Trap-Neuter-Return (TNR), we applied a framework requiring identification of areas of agreement, areas of disagreement, and identification of empirical data collection required to resolve disagreements. There is agreement that Australia has a problem with stray cats, causing problems of impacts on wildlife, nuisance, disease transmission (including public health issues and exchange of diseases between stray cat and pet cat populations), poor welfare outcomes for stray cats, and an emotional burden on staff euthanising healthy stray cats. There is disagreement on whether (i) current measures are failing, leading to unacceptably high euthanasia levels, (ii) some contributors to the debate misunderstand TNR, (iii) TNR trials will reduce urban cat populations and associated problems, (iv) TNR is an ethical solution to cat overpopulation, and (v) some contributors to the debate promulgated misinformation. Although not everyone agrees that TNR trials should proceed, as a hypothetical exploration, we propose an experimental approach explicitly comparing TNR to alternatives. Trials could only be considered if other detailed and well-funded attempts at stray cat control focusing across an entire Local Government Area (LGA) prove ineffective.

## 1. Introduction

Those following the exchange of views between Crawford et al. [1] and Wolf et al. [2] regarding the suitability of Trap-Neuter-Return (TNR) for managing stray cats in an Australian context will realise that debate is sharply polarised. Further statements of disagreement will do little to improve understanding of the issues unless alternative views are considered constructively to refine positions, exchange information, or agree on research programs to resolve outstanding matters with empirical data.

In this context, we applied the framework proposed by Kirkpatrick ([3] and refined by Kirkpatrick [4]) for proceeding with scientific debate on ecological issues to the TNR discussions. This requires declarations of interest by parties in the debate to ensure openness. This is followed by identification of the areas of agreement (it is rare for parties not to have common ground). Then areas of disagreement can be delineated, followed by dialogue concerning what further evidence might be collected to resolve the disagreements. Disagreement can also be compounded by differences in terms used to describe populations of cats, and so definitions need to be clear. The final step in the model is for proponents on both sides of the debate to describe the empirical data that would lead them to change their positions.

An expanded discussion of these points forms the body of this paper. The issue of how to name different populations of cats is considered first so that the terminology used throughout the paper is clear, followed by a brief exposition of the background to cat management issues in Australia. Subsequent points are structured under headings that follow Kirkpatrick’s framework.

## 2. What to Call a Cat?

As noted in the glossary in Crawford et al. [1], the text in Wolf et al. [2], and other discussions on cat management (e.g., [5,6,7]), many different terms are used to describe cats close to, and remote from, human habitation. Pet cats, housebound cats, domestic cats, stray cats, colony cats, feral cats, semi-feral cats, community cats, unowned cats and semi-owned cats are common examples (but ironically not clowder). The language chosen reflects human values and interests, and so the names themselves are not neutral but carry an emotional charge that can influence study design, reporting and reception, as noted in work on dogs [8]. In the case of cats, the language may colour perceptions and management options [9,10]. Here, we use terms following those in [5] and [6] that we believe reflect the ecological function of cats, specifically:

- *Feral cats*—these cats exist in self-sustaining populations usually remote from human habitation and therefore without any direct support from people. We do not consider their management in Australia in this paper but refer readers with an interest in this topic to the relevant Commonwealth of Australia *Threat abatement plan for predation by feral cats* [11] and the associated background paper [12].
- *Stray cats*—these cats live in association with humans, receiving partial support from people either directly through deliberate feeding or indirectly via scavenging refuse. These cats are also sometimes referred to as *‘semi-feral’.* Although we are comfortable with ‘stray cats,’ we note that some argue against using the term because of the implication that the cats were once owned, which may not be the case [13]. Stray cats will vary in their socialisation to humans, especially those that have never been owned. This affects their suitability for adoption.
- *Pet cats*—these cats belong to a household that claims ownership and provides the cats with food, shelter and veterinary attention while permitting the cats free access to the outside environment. In some instances, these are *‘housebound cats’* (*sensu* [6])—pet cats contained on their owners’ property—while in other instances they may be allowed to roam beyond property boundaries as *‘outdoor house cats’.*

From an ecological perspective, the survival, reproduction and behaviour of feral cats are independent of humans, whereas humans influence these points for stray and pet cat categories (a finer grained categorisation is given in Figure 1 of [14]). Behavioural differences such as hunting will also differ between the categories, which in turn may lead to different management approaches. We avoid the terms ‘community cats’ and ‘semi-owned cats’ because these imply a degree of wider social ownership or responsibility for stray cats that may not be shared by all citizens. We also avoid the term ‘domestic cat’ because this is the common name for the species *Felis catus* and can therefore lead to confusion when it is introduced into the literature on cat management because all populations of cats can be described using this term.

## 3. Cats in Australia—A Brief Background

Genetic evidence and historical records agree that cats spread across Australia following British colonisation in 1788 [15,16]. Deliberate release and straying from human habitation established feral populations across the continent and on the island state of Tasmania, aided by the spread of another introduced species, the European rabbit *Oryctolagus cuniculus*, as a food [17]. These feral populations now number between 1 and 11 million cats [18] and threaten at least 142 native species and sub-species (40 mammals, 40 birds and 21 reptiles) [12], are implicated in most of Australia’s >30 mammal extinctions since their arrival, and are the subject of a national threat abatement plan [11]. Australia is also home to c. 3.9 million pet cats [18,19], and c. 700,000 stray cats [18]. Management of pet cats and stray cats comes under the jurisdiction of state governments, with relevant legislation covering areas such as animal welfare, biosecurity, parks and reserves, and sometimes specific companion animal legislation. Legislation may also delegate some autonomy to local government. Thus, practices to manage stray and pet cats vary considerably across jurisdictions. Limited TNR management of stray cats is occurring, but is problematic under the law (e.g., [20]). Legal stray cat management involves trapping and impoundment in shelters, followed by either adoption or euthanasia. Management of pet cats commonly mandates desexing and microchip ID, with containment of cats to their owners’ properties required less frequently. The shifts in perceptions of cats with time in Australia are documented by Riley [21].

## 4. Declarations of Interest

While conflicts of interest are normally declared at the end of a paper, in cases of debate, we feel that a fuller disclosure in the main manuscript may be helpful for external parties, in accordance with Kirkpatrick’s protocol. In this context, we jointly declare that this work is not funded beyond the institutional support provided by our employer, Murdoch University. Calver is a life member of the Cat Welfare Society of Western Australia and a member of the Ecological Society of Australia, Crawford is also a member of the Cat Welfare Society of Western Australia, Torre Argentina (Roman Cat Sanctuary) and the Wildlife Preservation Society of Australia. Fleming is a member of the Australasian Wildlife Management Society. The opinions expressed in Crawford et al. [1] and this response paper are those of the authors and are not intended to represent those of the named organisations.

## 5. Areas of Agreement

Cats are Australia’s second most popular pet. An estimated 29% of Australian households own a cat and the national population of pet and housebound cats is estimated at 3.9 million [19]. It is more difficult to determine the number of strays, but one estimate is 300,000 in the state of Victoria alone [22]. Legge et al. [18] estimate 700,000 unowned cats (considering cats living in highly modified environments including urban areas but also rubbish dumps and piggeries) and 1.0 million to 11 million feral cats (including 95% confidence limits on the estimates). We agree with Wolf et al. [2] that the plight of stray cats cannot be ignored and that the problems they cause should be mitigated. Concern for the welfare of strays features in local news bulletins (e.g., [23]), raising substantial community concern. Many animals are processed through animal shelters where the outcome may be euthanasia, distressing the carers involved [24]. We also agree that neutering is important in population regulation. We believe that we have common ground with Wolf et al. [2] in acknowledging that stray cats also hunt wildlife, cause nuisance, may be a public health concern, suffer ill health themselves, and may transmit disease to wildlife or to pet cats; thus inaction is not an option. We may, however, differ in our assessments of the significance of these risks. 

## 6. Areas of Disagreement

In brief, Crawford et al. [1] argued that TNR is inappropriate in Australia because it is unlikely to lead to rapid, significant reductions in stray cat populations. Returning neutered stray cats to the environment may compromise their welfare [25] while at best making only modest responses to alleviate the problems of wildlife depredation, public health risk, nuisance and disease spread to wildlife and pet cats arising from stray cats. Instead, we proposed targeted adoption campaigns whereby, wherever possible, stray cats are neutered and then adopted rather than returned to the environment. This would be complemented by education campaigns to encourage neutering and containment of pets. Under this scenario only cats that could not be adopted would be euthanised. Wolf et al. [2] supported the adoption component of this approach but argued for the return to the environment, with the support of caregivers, of cats in good health that could not be adopted. We consider their arguments in detail below.

### 6.1. Are Current Adoption Measures Failing?

**Figure 1:**
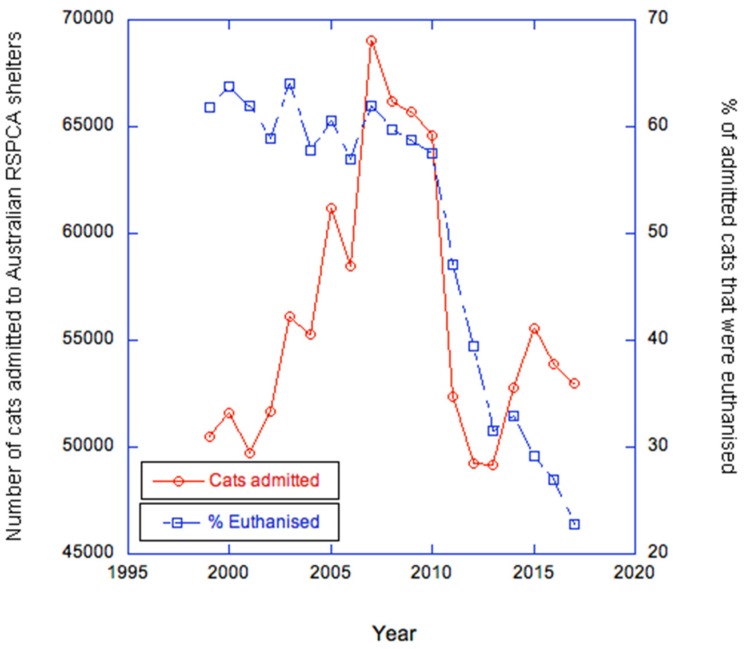
Cats admitted to Australian RSPCA shelters and % of those cats euthanised between 1999/2000 and 2017/2018. Source: RSPCA Australia (2019).

Wolf et al. [2] argued that TNR is needed urgently now because current adoption-based approaches are unsuccessful. They quote figures for Royal Society for the Prevention of Cruelty to Animals (RSPCA) shelters to show that admissions of cats were similar across the reporting years 2011/2012 to 2017/2018 and, on that basis, argue that current approaches are unsuccessful because admissions are stable. In fact, RSPCA records are available going back to 1999/2000 [26]. These show considerable fluctuation: admissions peaked at 69,034 in 2007/2008 and then declined to a low of 49,166 in 2013/2014, but rose again to 55,570 in 2015/2016. Numbers have been quite stable since, with 53,011 cats processed in 2017/2018 (Figure 1). We do not take a bleak view of the figures, for the following reasons. 

First, although there can be substantial variation across shelters, approximately half of admitted cats are strays, while the others are surrendered owned cats [24]. More specifically: ‘Based on RSPCA statistics nationally and in Queensland, of cats entering shelters, approximately half are kittens and half cats (53% kittens, and 47% adult cats over 2006–2010). Of adult cats, approximately 50% are surrendered owned cats and 50% are stray, mostly socialised to people (only 10% of cats entering shelters are categorised as feral and 92% of these are euthanized). Importantly, of kittens entering shelters, approximately 44% are from owned queens and 56% are stray (only 9% are categorized as feral).’ [27] (p. 7)

A significant component of the pressure on shelters arises from factors such as allowing owned queens a litter, some owners treating their cats as disposable assets surrendered when they are no longer convenient, problems with rental accommodation prohibiting pets, or owners surrendering cats because of their own illness or infirmity. The high proportion of stray cats socialised to people also suggests abandonment, poor ID preventing return of lost cats, or indifference of owners in searching for lost cats. Abandonment is difficult to quantify, but Kreisler et al. [28] noted that *c*. 18% of cats trapped in the long-running ORCAT TNR program in Florida, USA were already neutered. Excluding the 41% of these cats with tipped ears (indicating neutering in TNR activities), this left *c*. 11% of the cats as having been previously owned and either lost or abandoned. This would be a minimum figure, because it does not include any entire animals lost or abandoned. In the USA, when owners search for a lost cat their success is 53% and when someone finds a lost cat success in reuniting it with the owner is 38% [29,30]. In Australia, owners who search for a lost cat have a 61% success rate [31], while shelter data indicate that less than 5% of stray cats are reclaimed by owners [32].

Australian owners microchip cats at older ages than dogs [33], which may contribute to problems in reuniting lost cats with their owners. Therefore, several authors endorse collar-mounted ID to aid reuniting lost cats with their owners [34,35]. Abandonment and poor ID of owned cats are not addressed by TNR but resolving them will assist in reducing the numbers of cats surrendered and not reclaimed, reducing the pressure on shelters. 

Second, admissions have pulled back from the peak of 2007/2008. This could be argued as evidence that current approaches are making a difference. 

Finally, and most importantly for the context of the present discussion, euthanasia rates in shelters are falling significantly, from 61.8% in 1999/2000 to 22.8% in 2017/2018 (Figure 1). Even the Australian news media report that ‘the rate of cats and dogs euthanised in RSPCA shelters has dropped by approximately two-thirds since 2000. The percentage of cats being adopted or rehomed has doubled’ [32].

The reductions in euthanasia rates are the result of significant effort exemplified in the Australian Cat Action Plan (ACAP), developed by the Getting 2 Zero (G2Z) group of the Animal Welfare League of Queensland [27]. Through wide consultation, they have developed 12 strategies that together aim to reduce the flow of cats into the unowned cat population and to remove (and rehome where possible) unowned cats. Varying euthanasia rates across different Australian shelters are explained, at least in part, by the adoption of ACAP practices in shelters. For example, Kerr et al. [36] report a fall in cat euthanasia from 58% to 15% in Queensland RSPCA shelters from 2011 to 2016, with an impressive fall in the total number of kittens euthanised from 1116 in 2011 to just 22 in 2016. They attributed the fall in euthanasia to increased adoptions of kittens and poorly socialised cats, which were assisted by foster programs. Rand et al. [37] report that the range of euthanasia rates for cats admitted to Victorian council shelters in 2016–2017 varied from 6.5% to 83.9% (mean 48.3%), with councils employing a range of strategies aimed at reducing animal intake, while increasing reclaim and rehoming rates. Funding, sheer numbers of cats, and poor owner education were cited as barriers to reducing euthanasia rates. Although the ACAP does endorse a form of TNR (TDARS, trap-neuter-adopt-return-support), it does recognise that this should not be applied in ecologically sensitive environments and requires closed populations. Furthermore, TNR is problematic in all Australian states under legislation to prevent the abandonment of domestic animals (see [20] for the specific case of New South Wales), and so although limited illegal TNR programs may be occurring, the significant successes reported under the ACAP essentially occur in the absence of TNR. 

**Figure 2:**
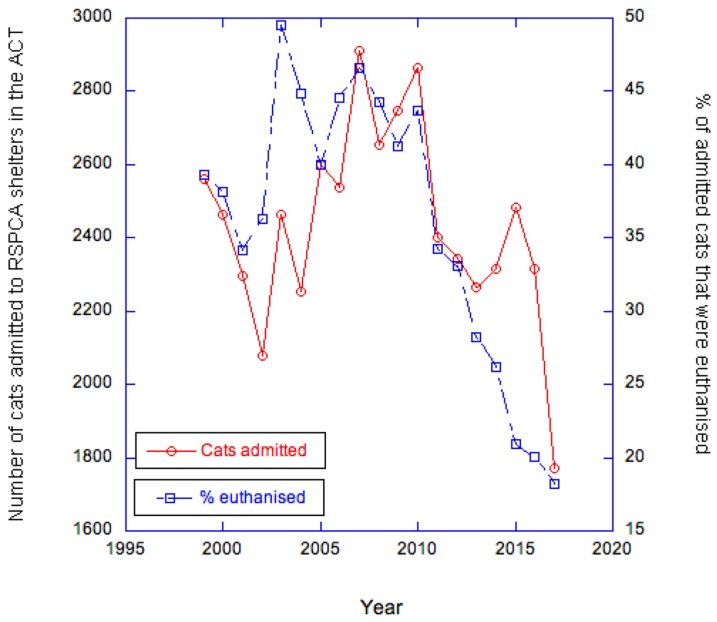
Cats admitted to RSPCA shelters in the Australian Capital Territory (ACT) and % of those cats euthanised between 1999/2000 and 2017/2018. Source: RSPCA Australia (2019).

A further example of stray cat control with an emphasis on trapping and adopting is described in the Australian Capital Territory’s (ACT) ‘Draft ACT Cat Plan 2019–2029’ [38], in which one of the strategies is ‘trap, neuter and adopt.’ It is unclear in the draft what should happen to animals that cannot be adopted (it may mean that they are insufficiently socialised to adopt, elderly or suffering from chronic health problems. Such cats are unlikely to be released under TNR either). Of course, a draft may vary from the final document, but we support the intent to remove stray cats from the environment. In this context, one would expect the declaration of cat containment areas in parts of the ACT under Section 81 of the *Domestic Animals Act 2000* to (i) possibly lead to a short-term increase in shelter admission of cats because some owners find this more convenient than complying, followed by (ii) an overall decline in shelter admissions as owners become more responsible and fewer cats are abandoned to swell stray populations. RSPCA data on shelter admissions in the ACT do show a drop from 2560 in 1999–2000 to 1773 in 2017–2018, which is consistent with such a prediction. Euthanasia is also declining (Figure 2).

We argue for strengthening support for the already successful measures in the ACAP, which are reducing euthanasia without the need to return neutered stray cats to the environment. Further funding and volunteer support of existing initiatives should strengthen success. Adoption is, of course, only part of the solution. It needs to be applied in conjunction with strong measures to increase responsible ownership, together with education programs targeted to the needs of individual communities to raise awareness and compliance (e.g., [39,40,41,42]). The suggestions of some authors to encourage positive behaviour change towards unowned cats and to use TNR to encourage positive views of neutering [43] are of limited value in Australia, where concern about the environmental effects of cats is high [44] and the problem is less one of promoting a positive view of neutering and more encouraging people to neuter their cats before puberty [45,46,47]. Such methods should both remove cats from the environment and reduce the flow of new cats into the stray population. Effective legislation is therefore needed, including provision for licensing, identification, neutering and confinement of pet cats, together with adequate funding for enforcement. The ACT, for example, has had a very positive experience with cat containment in some suburbs, with over 91% of ACT residents sampled (including 74% of cat owners) agreeing that there are benefits of cat containment, especially with regard to wildlife protection, reduced nuisance activities, and lower veterinary bills for owners [46]. Creation of an Australian equivalent to the UK’s Kitten Neutering Database (KiND, www.kind.cats.org.uk) would help greatly in encouraging prepubertal neutering of pet cats in Australia, as recommended by Crawford et al. [47]. These measures, together with owner education, should reduce the number of stray cats. 

### 6.2. Misunderstanding of the Purpose and Process of TNR

Wolf et al. [2] argue that we misunderstand TNR because TNR does not simply neuter and release stray cats—adoption is a significant part of TNR activities, as are veterinary treatments such as vaccination and worming when cats are held, and support of cats by feeding once released. In response, as outlined in the section above, adoption of stray cats has indeed been successful in reducing euthanasia rates. Furthermore, the review of literature we presented graphically (Crawford et al. [1]; Figure 1) clearly indicates that much of the success in population reduction reported by published TNR studies available to us at the time had been due to adoption. The ‘R’ in TNR indicates that this option *releases* unadopted animals (generally back to their point of capture), which is our point of contention with the application of TNR.

Wolf et al. [2] also argue that stray cats are present already so that TNR does not add to the number of cats in the landscape and, therefore, there will be no increase in cat densities. Density is a function of population and area, and so if cats concentrate around feeding stations, localised densities will increase. TNR does provide post-release caregiving and we wish to emphasise the scale of the support required from caregivers if all the unadoptable cats currently entering shelters were instead supported on the streets. In Crawford et al. [1], we simply calculated how many supported colonies would be needed to include the approximate numbers of stray cats that need to be considered. The estimated numbers of 311 new colonies per year cited in Crawford et al. [1] assume that each of the 3640 cats processed annually through the RSPCA shelters identified with behavioral/temperament issues (making their adoption unlikely) is released into a colony of *c.* 11.5 cats (based on the median colony size reported in [48]) that would then require caregiver support.

It is also important to understand the legal context of proposed TNR in Australia. While legislation varies from state to state, there are varying degrees of provision against abandonment or feeding under animal welfare or biosecurity acts. For example, in Queensland, unowned cats (defined as *Felis catus* and *Prionailurus bengalensis x Felis catus*) are listed in categories: 3 (must not be distributed into the environment), 4 (must not be moved) and 6 (must not be fed (except to trap)) of the *Queensland Biosecurity Act 2014*. In New South Wales, releasing a neutered cat might contravene the *Prevention of Cruelty to Animals Act 1979* that prohibits abandoning an animal, or the *National Parks and Wildlife Act 1974* that prohibits releasing a non-native animal anywhere in New South Wales [20]. In this context, TNR colonies need to be ‘established’ even when the cats were present originally, because releasing neutered cats back into the environment, or in some cases even feeding them, can incur potential prosecution under current legislation. On a final note, we have also argued that providing a focal point where cats are fed will encourage abandonment by irresponsible owners, a problem noted in TNR studies elsewhere (e.g., [49,50]).

### 6.3. TNR Will Reduce Urban Cat Populations

Wolf et al. [2] state that TNR is likely to lead to reductions in stray cat populations and that we have ignored important data that support this stance. Wolf et al. [2] state that ‘Conspicuously, ‘Letting the Cat Out of the Bag’ fails to mention two Australian TNR studies demonstrating a 30% reduction in cat numbers over 2 years [48], and a 50% reduction over 5 years [51].’ Both these studies are cited in our original paper, with data from [51] included in Figure 1 and Table 1 in that paper. Data from [48] were not included in the figure and table because they did not meet the selection criteria of an initial census before TNR commenced matched with a follow-up census.

Our point in relation to these studies and to others claiming population reductions following TNR remains that a large component of claimed reductions is removal of cats by adoption, euthanasia, migration, or death in situ. For example, of the 195 cats in one program, 59 were adopted, 67 disappeared, six were euthanised, three were returned to their owners, 13 died from other causes, two were relocated and one was seized [52]. In the ORCAT study in Florida, USA, 1111 cats were returned, but 1419 were removed by adoption, transfer to an adoption centre, euthanasia, death in care or dead on arrival [28]. One of the lowest adoption figures on record is 15%, but this was under the premise that ‘Original protocol called for all free-roaming cats without serious illness or injury to be returned to locations of capture ... however, over time, as feline intake declined and more shelter space became available, some sociable cats were admitted for adoption or transferred to rescue groups’ [53] (p. 4). We contend that the population reductions reported in TNR can be achieved without returning cats to the environment. 

### 6.4. TNR Will Reduce Problems Associated With Urban Cat Populations

Wolf et al. [2] suggest that we have exaggerated the potential problems caused by returning neutered cats to the environment. We argue that if the presence of stray cats requires intervention, then returning neutered animals to the environment is simply not addressing many of the core problems, especially in relation to wildlife protection and spread of disease.

We acknowledge that there are differing interpretations of the evidence that predation by pet cats or stray cats can cause declines of native species; as Wolf et al. [2] point out, one of us has been cautious regarding this point before [54]. Nevertheless, we believe that there is growing evidence, including some compelling recent Australian examples, that predation by pet or stray cats is a significant problem (Table 1). Some of these cases include local extirpation, which can happen rapidly and therefore be hard to detect. The increasing weight of evidence no doubt underpins the ACAP recommendation that TDARS not be implemented in ecologically sensitive areas [27].

With regard to public health (and, on occasion, health of companion and agricultural animals), it is well-established that cats carry a range of species-specific and zoonotic diseases and parasites that can be a concern to public health, the health of companion animals or wildlife, and agricultural production (e.g., [55,56], [57], chapter 8). For example, in a consideration of human seroprevalence to the cat-borne parasite *Toxoplasma gondii* on Mexican offshore islands, the authors concluded that eradication of cat populations, not control of them, could be a public health benefit [58]. Woinarski et al. ([57], chapter 8) provide an extensive review of the distribution of *T. gondii* in Australia, including estimates of human health costs, while Stelzer et al. [59] assess costs for livestock production internationally, including Australia.

Concerns relate not only to pathogens already present in Australian cat populations (such as *T. gondii*), but also to those that could be introduced. Thus, although rabies is not present in Australia, its spread in eastern Indonesia increases the risk of entry to northern Australia [60] and has prompted calls for rabies vaccination of Queensland veterinary clinical staff and students [61]. Responses would involve management of a range of susceptible domestic animals, including cats [62]. We therefore argue that control of stray cat populations is required for management of those diseases already present in Australia and to reduce potential reservoirs of hosts for introduced diseases. Preferably this should be by removing these animals, not by neutering them and returning them to the environment where they continue to contribute to the problems and where they can be inaccessible to veterinary care, should it be required. 

We agree with Wolf et al. [2] that the mental health stress on staff who euthanise healthy companion animals in shelters is a substantial issue. While not intending to belittle these stresses, we do wish to acknowledge the complexity of the issues explained in the original sources. The full quotes are (i): ‘Additionally, research has indicated specific characteristics of the profession likely contribute to a greater than expected number of deaths from suicide among veterinarians, including long work hours, work overload, practice management responsibilities, client expectations and complaints, *euthanasia procedures* (our italics), and poor work-life balance.’ ([63], p. 109); and (ii) ‘Other factors likely include financial debt and knowledge and acceptance of *euthanasia procedures* (our italics) as well as access to potentially lethal pharmaceutical products. Additionally, veterinarians are trained to view euthanasia as an acceptable method to relieve suffering in animals, which can affect the way veterinarians view human life, including a reduced fear about death, especially among those experiencing suicidal ideation.’ ([63], p. 110)

In Australia specifically, Jones-Fairnie et al. [64] raised concern about suicide rates in veterinarians. A more detailed follow-up study noted: ‘ ... disturbances in psychological well-being are a major problem in the veterinary profession. Causes include professional issues, such as dealing with difficult or upset animal owners, and the emotional issues surrounding animal euthanasia. In addition, most veterinarians are managing their own small business and therefore are dealing with issues such as finances, staff and regulatory requirements. Further, many veterinarians work long hours and the difficulty of recruiting locums limits holiday opportunities. In rural areas, these difficulties are compounded by professional isolation and lower remuneration because of the rural economic depression.’ ([65], p. 76)

Euthanasia is not the only matter affecting mental health in veterinary professionals. Current strategies for controlling stray cats in Australia are reducing euthanasia of healthy animals, and so they are part of the solution. We argue that an integrated approach to removing cats from the streets and encouraging containment of pet cats should reduce euthanasia of healthy animals and the prevalence of trauma.

### 6.5. Is TNR An Ethical Solution to the Problem of Cat Overpopulation?

Wolf et al. [2] fairly challenge us to explain our ethical stance on TNR. To set context, there are claims of improved stray cat welfare following neutering, release, and subsequent supplementary feeding based on factors such as visual appearance of cats [76], increased longevity [28], reduced prevalence of infectious disease [28] or skin lesions [77], and reduced fighting [76,78]. Against these must be balanced the acknowledged risks of the free-roaming lifestyle for all cats, irrespective of ownership status, such as: predation [79,80], human persecution [81], accidental poisoning or ingestion of other hazardous substances [25], accident trauma [82], and fighting injuries [82]. It is for these reasons that animal welfare bodies such as The Humane Society of the United States (HSUS) [83] and PETA [84] endorse containment of pet cats on their owners’ properties. While HSUS does support TNR for stray cats, PETA takes what we consider the more logical position of endorsing containment of pet cats but not TNR except under very specific conditions because of the hazards to free-roaming cats [85,86]. The likely effect of TNR on problems such as wildlife predation, public health, disease transmission to wildlife and pet cats, and public nuisance should also be considered alongside cat welfare.

A range of ethical positions relating to companion animals that could apply to TNR is summarised in Table 2. There is no right or wrong in these, but actions can be explored for consistency with particular approaches.

Our position corresponds with utilitarian/consequentialist approaches, which strive to achieve the best consequences overall for all sentient beings involved. We argue that stray cats present a range of problems that justify intervention, including predation on wildlife, disease transmission, and poor welfare of the cats themselves. We therefore favour targeted adoption with euthanasia of animals that are unsuited to adoption. We argue that the overall benefit of such an approach outweighs the euthanasia of individual cats. The adoption approach also provides opportunities for people to work closely with cats as expressions of their emotional attachment, as evidenced by media reports on people dedicated to socialising cats for adoption ([87,88]). It also avoids exposing released cats to the hazards of life on the streets, which are behind the opposition of the animal welfare group PETA to TNR (e.g., [85]), or at best the use of TNR only under very specific conditions [86]. PETA’s position corresponds closely to our own: ‘Having witnessed firsthand the many gruesome fates of homeless cats and of the animals they prey upon, PETA believes the ideal solution lies in preventing cat homelessness in the first place, by passing and enforcing strong responsible guardianship laws, including requirements that cats (and dogs!) be spayed and neutered, licensed and microchipped, and kept indoors or safely contained on their guardian’s property. Homeless cats need—and deserve—to be brought indoors or taken to open-admission animal shelters where they are safe and warm and have a chance at being adopted into loving homes’ ([86], pp. 1–2).

If the targeted adoption approach is combined with steps to reduce cat abandonment and responsible management of owned cats, including containment of cats on their owners’ properties, many problems would be resolved. We do not believe that TNR advocates would similarly adopt the utilitarian/consequentialist position, instead tending to one or more of the following approaches.

Deontological and rights approaches, contextual approaches, relational approaches and compassionate conservation can be invoked in support of TNR, with the fundamental arguments being that euthanasia of stray cats violates their right to life, some people have deep emotional involvement in the welfare of stray cats, and that the virtue of compassion precludes intentionally causing harm. We find five main problems in common across these approaches.

First is balancing the right of the individual cat against that of the animals that it kills or infects with pathogens. Note that this is not a population argument, it is about the relative value of a cat’s life versus that of its prey. Under a relational approach the justification would be a special duty to cats as companion animals, but this is not a position we share.

Second is whether or not neutering a cat and depriving it of reproductive behaviour and experience is in the best interests of the individual cat itself. As Sandøe et al. [89] observed, ‘On almost all accounts on which animals have rights, neutering is ethically problematic’. They also note that under contextual approaches neutering can still be problematic, because decisions may be dependent on individual relationships and could be interpreted as dominating. Thus, in TNR, there is a contradiction between the argument for no euthanasia of healthy animals (rationalised as animal rights) and the need to neuter cats (in contravention of their rights).

Third, in the wider context of human relationships with animals, it is necessary to demonstrate why cats particularly are singled out for a TNR approach as opposed to other animals. The relational argument that cats are companion animals does not sit easily here. Rats, for example, are also valued human pets and, when socialised, appear to take pleasure in interactions with people [90], yet lethal control of rats is common practice. An outline of strategies to reduce stray dog populations and associated euthanasia in Australia highlighted desexing programs (low cost or free), encouragement of tagging and microchipping, and reduction of problems that contribute to surrender of owned dogs (e.g., boarding, feeding assistance, training, rental restrictions on pets)—all points that we advocate for cats—but not TNR [91]. Why should cats be treated differently?

Fourth, if one accepts that we do have a greater duty to domestic animals, TNR then needs to justify how that greater duty is met by returning cats to a risky life on the streets. Finally, many of the predation and disease problems represented by stray cats are not solved by neutering them and returning them to the environment. If, as Wolf et al. [2] imply, these problems are insignificant, then why is action needed at all?

### 6.6. Misrepresentation of Research

One section of Wolf et al. [2] is devoted to the claim that: ‘A significant amount of the research cited in ‘Letting the Cat out of the Bag’ is misrepresented, often as a result of what has been called “daisy-chaining”, the practice of citing authors who are “merely repeating [material] from what an earlier publication cited” ... We document these claims of misrepresentation in Table 3, responding to each by returning to the original sources and, where necessary, quoting from them. Readers may draw their own conclusions. We stand by our original statements.

## 7. What Evidence Would Cause Us to Change Our Minds?

To complete the steps of Kirkpatrick’s model, it is necessary for us to state the data that would cause us to change our minds—to accept that TNR should be trialled in an Australian context. On the basis of the international literature, we are doubtful that TNR would make a significant difference to stray cat numbers in Australia, or that it would mitigate associated problems. We argue that a well-funded program of trapping and removal, with a strong focus on adoption, would be more likely to succeed. Data that would lead us to change our minds could come from two sources: research on the problems (or lack of them) caused by stray cats in Australia and the outcome of biosecurity planning in Queensland.

### 7.1. Research on Stray Cats in Australia

Lack of Australian data on stray cats is an issue that hinders development of an argument for or against TNR. Knowledge of the diets of stray cats, their health and life expectancy, and the prevalence of pathogens and parasites of zoonotic concern should all be considered, building on the literature review of Denny and Dickman [106] and recent empirical work [25]. A minimum requirement for a trial would be data from multiple locations demonstrating that stray cats are not a risk to wildlife (including potential reintroductions of wildlife to suburban bushland or even gardens), that zoonoses are of minimal risk, and that stray cats do not suffer trauma or disease at greater levels than pets. It would also be useful to know the outcomes for rehomed stray cats (relinquishment, behaviour problems). Studies of these prerequisites are currently sparse, but some work has been done [107].

### 7.2. Biosecurity Planning in Queensland

Under Section 53 of the *Biosecurity Act 2014*, local governments in Queensland ‘... must have a biosecurity plan for invasive biosecurity matter for its local government area.’ The Brisbane City Council’s Plan (Brisbane City Council 2018) includes provision for managing unowned cats within the Brisbane Local Government Area (LGA) that includes the specific operational objectives:

- To remove non-domestic cats from areas where they pose risks to native biodiversity.
- To reduce non-domestic cat numbers in other situations, particularly where they have or could have environmental or social impacts.
- To educate the community about the impact of non-domestic cats on the natural environment.
- To educate the community about responsible pet ownership [108].

The plan began in February 2018 and runs until 31 December 2022. Assuming that there is active pursuit of these objectives (including the educational ones), we would accept that failure to eradicate (or at least significantly reduce numbers) of non-domestic cats from areas where they pose risks to biodiversity and failure to reduce their numbers in other situations where control was implemented by 31 December, 2022 would be an indication of lack of success of a removal approach.

### 7.3. Hypothetical—Design of a TNR Trial in Australia

As a hypothetical step, we are willing to consider the design of a TNR trial in Australia, which should only proceed if the criteria outlined above are met and adequate long-term funding is available. Key steps would be:

1. Any trial needs to compare alternative approaches, with a Before-After-Control-Impact (BACI) design [109] a strong model for evaluating TNR. For us, one key question is not simply whether or not TNR leads to population reductions, but how the magnitude and speed of any such reduction compares to other options such as the targeted adoption approach we espouse, or to lethal control. A trial would therefore need to identify multiple sites, which would be randomly assigned to treatments: we suggest TDARS versus trap and remove (involving adoption plus euthanasia of unadopted animals) versus lethal control, with control sites that are not manipulated at all.
2. Sites should be chosen with minimal likelihood of environmental impacts caused by returned cats. This would require close collaboration with wildlife societies for specialist advice (e.g., Birdlife Australia). It may also be difficult to achieve this goal, because even where prey are common, the site may still function as a population sink for prey [110]. Ideally, there should be an environmental impact assessment (EIA) at proposed sites.
3. Wildlife should be censused before any intervention to give reliable baselines for changes. This should include native and introduced species. Monitoring should continue throughout the trial.
4. Cat numbers should be censused before any intervention. Any change in population size can only be measured against a robust baseline.
5. A wide range of dependent variables should be considered for monitoring, including: changes in populations of wildlife, numbers of cats processed (including euthanasia and adoptions), cat population census, health status of handled cats, admissions to local animal shelters, and complaints about cats to local councils. It is critical to have data on all these variables. Data on shelter admissions, for example, are unhelpful alone—if cats are neutered and released rather than admitted, simply instituting TNR must reduce shelter admissions. However, if reduced admissions tally with reduced nuisance and no changes in wildlife, then they indicate a suite of positive responses.
6. Accurate costs should be kept for comparison between treatments.

Now that we have outlined the data that would cause us to change our minds on whether or not to proceed with a TNR trial and what shape that trial should take, we invite advocates of TNR trials in Australia to indicate what data would lead them to shift their position.

## 8. Conclusions

We believe that almost all parties to the debate over the management of stray cats in Australia would agree that the pressing problems of cat welfare, wildlife depredation, nuisance, public health, and health of pet cats and other domestic animals require action. Our position, based on a review of the literature, is similar to that of Castro-Prieto and Andrade-Nunez ([74], p. 110), who noted that: ‘TNR was ineffective in addressing other impacts associated with large populations of stray cats, including predation, diseases, and odors from the cats’ urine and feces in public areas.’ We advocate instead for removal with a significant component of targeted adoption, accompanied by a wider social effort at encouraging responsible cat husbandry. This is consistent with utilitarian/consequentialist approaches to the ethics of cat management, while avoiding the need to advance the rights of individual cats over those of other sentient animals with which they interact, justify neutering in view of its disregard for a cat’s right to reproduce, and to justify why cats should be treated differently to other sentient vertebrates managed by humans. It also prevents the welfare problems associated with returning cats to the streets and places responsibility squarely where it belongs—on responsible ownership involving neutering pet cats, containing them on their owners’ property, and requiring licensing and ID to facilitate return of lost animals and to discourage abandonment.

## Figures and Tables

**Table 1:** Examples of significant predation pressures by pet or stray cats on wildlife in Australia.

Prey taxon	Impact	Reference
**Reptile**		
Bluetongue lizard *Tiliqua scincoides*	Predation on juveniles in outer suburbs of Sydney, New South Wales, was regarded as significant by the authors.	[66]
Olive legless lizard *Delma inornata*	Predation by one pet cat reduced the local population to a non-detectable level.	[67]
Skink *Ctenotus fallens*	A suburban garden population was extirpated by a single pet cat.	[68]
Reptile species in south-eastern Australia, based on records of the Wildlife Information and Rescue Service (New South Wales 1989–1998)	Cat attacks were responsible for *c.* 3% of all snakes and *c.* 8% of all lizards rescued, with smaller species or juveniles of larger species most likely to be attacked.	[69]
**Bird**		
Superb lyrebird *Menura novaehollandiae*	Predation of young birds in Sherbrooke Forest, Victoria.	[70]
Splendid fairy-wren *Malurus splendens*	Over a 15 year study in the Perth hills, Western Australia, c. 10% of nests were destroyed by cats.	[71]
Fairy tern *Sternula nereis nereis*	Predation by a single stray, neutered cat and one pet cat caused the total breeding failure of a colony.	[72]
**Mammal**		
Eastern barred bandicoot *Perameles gunnii*	Cat predation caused 17.8% of mortality of *P. gunnii*, mostly juveniles and sub-adults	[73]
Eastern ringtail possum *Pseudocheirus peregrinus*	Cats killed 37% of 57 radio-collared possums in Manly Dam Reserve.	[67]
Feather-tailed glider *Acrobates pygmaeus*	A local NSW population was extirpated by one pet cat.	[74]
**All Terrestrial Vertebrates**		
Terrestrial vertebrate species reported to the Bonorong Wildlife Rescue Service, Tasmania, a 24 h state-wide wildlife rescue service, between June 2010 and December 2016	‘Suspected toxoplasmosis’ and ‘Cat attacks’ comprised 5.93% and 5.69% of all calls respectively, making them the fourth and fifth largest categories of calls after ‘Road trauma,’ ‘Orphan’ and ‘Unable to fly.’	[75]

**Table 2:** Common ethical approaches to companion animal ethics, adapted from Sandøe et al. (2015), Hampton et al. (2019) and Animal Ethics Dilemma (undated). Alternative classifications are possible.

Approach	Description	Implications
Utilitarian/consequentialist approaches	Seek to obtain the best consequences overall for all sentient beings involved in a particular situation/interaction. The consequences are prioritised over other ethical concerns. In the context of conservation, the aim is ‘to ensure that the best possible animal-welfare outcomes are achieved and that they align with conservation priorities’ [92].	Ethically problematic actions such as killing can be justified if the positive consequences overall exceed the negative consequences.
		Our position is that the negative consequences of killing stray cats that cannot be adopted is justified by protection of wildlife, reduction of public health risk, reduction of public nuisance (including disease transmission and fighting involving pet cats) and prevention of poor welfare outcomes for the cats themselves. Given the negative consequences of returning neutered cats to the environment, TNR would be ethically problematic.
Contractarian approaches	Animals only matter if, and to what extent, people accept that they matter (e.g., [89,93,94]).	By excluding animals from a moral contract, there is no moral problem in animal suffering (though there would be if an animal’s suffering caused distress to people).
		If invoked to support TNR on the basis of distress caused to people by the suffering of stray cats, the challenges would be to demonstrate (i) that stray cats returned to the streets did not experience negative welfare outcomes that would distress people, (ii) that predation by TNR cats did not distress people who value the outcome for the prey, and (iii) that problems such as disease transmission to pets, fighting and nuisance did not continue to cause distress to people because cats are left in the environment.
Relational approaches	These focus on human relationships with animals and with other humans. Key aspects are that humans have greater duties to animals depending on how close animals are to us, and that we should be concerned about how we treat animals because this may influence how we treat fellow humans [94].	From this perspective, cats, as domestic animals, deserve better treatment than wildlife or pest species, or that given that cats are sentient humans have responsibility for them where cats and people co-exist [95].
		We believe that the position is compatible with TNR, given that it prioritises the welfare of cats over those of wildlife that may be threatened by TNR cats. The challenges, though, are to justify why (i) TNR is adopted for cats and not for other stray domestic vertebrates such as dogs or escaped aviary birds, and (ii) how returning cats to a dangerous life on the streets is fulfilling our greater duty to them as domestic animals.
Deontological and rights approaches	It is not only consequences that matter. There are intrinsic animal rights that must be respected and that creates duties and obligations of people towards animals [89].	Under this approach, it can be argued that animals have a right not to be killed, and so killing would not be ethical.
		The challenges in a TNR context are to (i) balance the right of cats not to be killed with the right of wildlife not to be subject to predation by cats in TNR colonies, (ii) justify neutering cats and removing their rights to a wide range of hormonally-mediated behaviour, including reproduction, (iii) give a rationale for neutering pregnant queens and thereby killing the foetuses, (iv) justify why cats specifically are singled out for such an approach rather than all unowned domestic vertebrates, and (v) explain how TNR accounts for the right of stray cats not to suffer given the poor welfare outcomes for many stray cats, including those in TNR programs.
Contextual approaches	Human relations to animals occur in a context which can take the form of intrinsic virtues, agreed commitments, or emotional involvement and consequences. The context takes primacy over any absolute moral rules or evaluation of consequences of actions [89].	The deep emotional involvement of TNR participants in the welfare of stray cats could be argued as justification for proceeding with TNR, irrespective of any negative consequences that may follow.
		The challenges in a TNR context are identifying (i) on-going responsibility for feeding and maintaining TNR colony cats, and (ii) how we can simultaneously commit to urban wildlife conservation (with the deep emotional commitment this invokes in some people) as well as stray cats (given the threats TNR cats may pose to wildlife) (see discussion in [96], Chapter 14).
Compassionate conservation	Develops from the contextual approaches above. Avoids deliberately harming animals, including harm inflicted in the interests of conservation such as culling one species to advantage another. Draws on the long-standing tradition of virtue ethics, where character traits deemed to be virtuous direct ethical conduct [89,92].	Compassion is seen as a virtue, and so if it is applied to conservation, it would preclude intentionally harming animals to achieve conservation goals. Under such an approach, euthanising cats would not be seen as an ethical solution to protecting wildlife.
		The challenge in a TNR context is that release of animals into high-risk environments where they are not reliably fed or cared for harms them indirectly.

**Table 3:** Responses to claims of misinformation in Crawford et al. (2019a).

Claim of Misinformation	Response
‘For example, the claim that “human exposure to rabies is more commonly caused by cats than other domestic animals” fails to acknowledge that the underlying data were compiled during an outbreak of the raccoon variant in the Mid-Atlantic region of the US. And as one of the original sources ... reveals, the pattern is reversed in other parts of the country, with dog exposures outnumbering cat exposures, as has been documented elsewhere.’	While we do not doubt the specifics raised by Wolf et al. [2], as a generalisation the statement that most cases of rabies exposure in the US from domestic animals come from cats is repeated in a more recent source [55], which also notes that in the US cats are responsible for approximately one-third of human post-exposure treatment for rabies. For purposes of rebuttal we quote: ‘Since 1988, rabies has been detected more frequently in cats than dogs in the United States …, and in 2008 the number of rabies cases in cats (n = 294) was approximately four times the number of cases in dogs … In 2010, rabies cases declined in all domestic animals, except for cats, which comprised 62% (*n* = 303) of all rabies cases in domestic animals … In contrast, dogs accounted for 69 rabies cases, which is a 14% decrease from 2009. Although rabies is detected most frequently in various wild animals in the United States and the majority of human rabies cases in the United States are attributable to bites of rabid bats, multiple studies have disclosed that human exposure to rabies is largely associated with free-roaming cats because of people being more likely to come in contact with cats, large free-roaming cat populations and lack of stringent rabies vaccination programmes …’ ([55], p. 190) Turning to one of the sources cited by Wolf et al. [2], the complete abstract of [97] states: ‘During 1993-2002, cats accounted for 2.7% of rabid terrestrial animals in New York but for one-third of human exposure incidents and treatments. Nonbite exposures and animals of undetermined rabies status accounted for 54% and 56%, respectively, of persons receiving rabies treatments.’ The number of exposure incidents cited for cats (4266) was therefore larger than any other species (next closest was raccoons (3298); the dog was the next closest domestic animal (3052) ([97], their Table 2). We do not consider that our original statement is a misrepresentation.
‘the claim that “Jessup ... documents studies from the USA that note reduced populations of native bird species, including complete absence of ground foraging species, near sites where unowned cats were fed” is contradicted by the results documented in the original sources ...’	The peer-reviewed original source is Hawkins et al. [98], from which we quote: ‘The presence of cats at artificially high densities, sustained by supplemental feeding seemed to reduce the abundance of native rodent populations, change rodent species composition, and may have facilitated the expansion of the house mouse. Bird numbers were also lower where cats were fed, and some species, such as California quail, may have been excluded completely from the areas with high cat densities.’ ([98], p. 32) Figure 1 in this paper presents the finding graphically. In our opinion, the statement in Crawford et al. (2019a) is supported by the original source.
‘In addition, a review of the sources provided to support the claim that “in some studies, poor physical conditions of TNR cats are easily recognizable” ... revealed no such evidence; the stray cats observed were not under TNR management.’	In the case of [23], it is true that the cats in question are not under TNR management and we thank Wolf et al. [2] for the correction. However, we do note that the cats are fed by a group called Community Cat Carers, who are also calling for donations to support neutering of the cats. Our point is that providing support for cats returned to the environment will not necessarily ensure health and good welfare (these cats are already fed, yet their health is poor). In the case of Castro-Prieto and Andrade-Nunez [99], we note that a TNR program was running in Old San Juan during the study. We quote: ‘Since then, both a local non-governmental organization called Save a Gato (SaG) and a cat shelter were established to advocate for the welfare of the cats under discussion; and started implementing TNR as a strategy to reduce and control the population of stray cats in Old San Juan. As a means of controlling the stray-cat population in this neighborhood, a program that combined TNR and adoption was implemented 10 years ago, but it remains unknown how effective that program has been’ (99], p. 111). The authors state that 32 cats (21%) of the population were in poor condition in the area of the TNR study (including near feeding stations) and include photographs. One cat photographed has an ear tipped, indicating neutering in a TNR program (assuming it is not a natural injury). Even discounting that, with 70% of the stray cat population identified as neutered by ear-tipping and 21% of stray cats in visibly poor condition, it is highly unlikely that no neutered cats were in poor condition. We believe our statement is justified.
‘More curious is one of the citations used to support a claim that feline immunodeficiency virus (FIV) “is commonly reported in stray cats.” Luria et al. actually reported “similar or lower prevalence rates of infections [in cats admitted to a TNR program] than those published for pet cats’ in the US.” ’	The claim implies that we used Luria et al. [100] to support our statement that FIV is commonly reported in stray cats. We didn’t. We cited the more recent review by Hosie et al. [101]. They note that: ‘... the seroprevalence of FIV is highly variable between regions, with estimates of 1–14% in cats with no clinical signs and up to 44% in sick cats.’ [101], p. 576). They also state: ‘Feline immunodeficiency virus is an important consideration in rescue shelters as the prevalence of infection is particularly high in populations with a feral background and in male cats. The prevalence may be lower in pre-owned cats that have recently been relinquished, compared with sheltered stray cats’ ([101], p. 581). In our opinion, the statement in Crawford et al. [1]—namely that FIV is commonly reported in stray cats—is supported by the source we cited. Furthermore, the comment about high variability between populations mentioned in Hosie et al. [101] explains why regional studies such as Luria et al. [100] may show high or low prevalence of FIV. For the record, we did cite Luria et al. [100] as evidence for the statement that ‘Adult males are more likely to contract the virus when competing for territory, females and food’ ([1], p. 14).
‘Also curious is what is missing from ‘Letting the Cat Out of the Bag’. It is noted, for example, that *Toxoplasma gondii* has “been detected in stray, pet and feral cat populations in Australia” and the article warns that “implementing TNR programs may facilitate proliferation of Toxoplasma”. This statement fails to consider, however, that older cats are more likely than younger cats and kittens to be immune as a result of previous exposure to the parasite and thus less likely to shed oocysts.’	We do not dispute the data regarding the influence of cat age, health and nutrition in spreading oocysts of *Toxoplasma gondii*, but the studies cited by Wolf et al. [2] also note: ‘In stray cats the pooled seroprevalence of *T. gondii* infection was significantly higher than in pet cats. This is consistent with studies reported in Spain ... Tehran ... and Brazil ... This higher seroprevalence in stray cats may be associated with their hunting and diet habits, as a stray cat lives outdoors, hunts and potentially feeds on oocyst contaminated scraps and garbage and/or *Toxoplasma*-infected wild birds and rodents, with more risk of ingestion of the parasite.’ ([102], p. 9). ‘Feral cats had the highest prevalence of infection.’ ([103], p. 1068). ‘If a cat owner knows about *T. gondii* but allows a cat to go outside, become infected, and shed oocysts, this action can be considered as intentional contamination of the environment with a potentially lethal zoonotic pathogen. If feline infections were better prevented, fewer oocysts would be added to the environmental reservoir, especially near human settlements. Our results support the recommendations … of not allowing cats to roam free and hunt prey. Preventing feline *T. gondii* infections is important to protect cats from clinical toxoplasmosis … and to protect public health.’ [104]. Our original point was simply that there is greater risk reduction associated with removing cats from the environment than in maintaining them there with food supplementation that is likely to attract other cats and encourage dumping. The studies cited by Wolf et al. [2] confirm the risks and add the charge that allowing a cat to roam freely is ‘...intentional contamination of the environment with a potentially lethal zoonotic pathogen’ [104]. In this context, De Wit et al. [58] make the case that eradicating cats on islands is likely to have public health benefits through decreased exposure to *T. gondii*. We believe that our original statement is justified.
Wolf et al. claim: ‘We agree with Crawford et al. that “capturing, transporting, neutering, vaccinating, worming and medicating are stressful procedures even for well-socialized pet cats, let alone for stray cats unsocialized/partially socialized to human contact”. However, the article fails to acknowledge that the authors’ alternate method would expose cats to the same stresses and more. … Yet the relationship between intake, length of stay, stress, infectious disease and euthanasia is not considered, nor the potential stress-related consequences of the proposed “targeted adoption” approach.’	We acknowledge that the stresses experienced by trapped cats are similar regardless of outcome, and we agree that under targeted adoption cats would remain longer in shelters (which must also occur under the adoption provisions of TNR programs). Our point is that the negative welfare consequences and other problems caused by returning cats to the environment justify the attempt to rehabilitate, foster and adopt as many as possible and to euthanise those that cannot be adopted to prevent prolonged shelter housing and related illnesses (e.g., [105]).
